# Rearranged EML4-ALK fusion transcripts sequester in circulating blood platelets and enable blood-based crizotinib response monitoring in non-small-cell lung cancer

**DOI:** 10.18632/oncotarget.6279

**Published:** 2015-11-02

**Authors:** R. Jonas A. Nilsson, Niki Karachaliou, Jordi Berenguer, Ana Gimenez-Capitan, Pepijn Schellen, Cristina Teixido, Jihane Tannous, Justine L. Kuiper, Esther Drees, Magda Grabowska, Marte van Keulen, Danielle A.M. Heideman, Erik Thunnissen, Anne-Marie C. Dingemans, Santiago Viteri, Bakhos A. Tannous, Ana Drozdowskyj, Rafael Rosell, Egbert F. Smit, Thomas Wurdinger

**Affiliations:** ^1^ Cancer Center Amsterdam, Department of Neurosurgery, VU University Medical Center, Amsterdam, The Netherlands; ^2^ Department of Radiation Sciences, Oncology, Umeå University, Umeå, Sweden; ^3^ ThromboDx B.V., Amsterdam, The Netherlands; ^4^ Translational Research Unit, Dr, Rosell Oncology Institute, Quirón Dexeus University Hospital, Barcelona, Spain; ^5^ Pangaea Biotech SL, Barcelona, Spain; ^6^ Department of Neurology, Massachusetts General Hospital and Neuroscience Program, Harvard Medical School, Boston, MA, USA; ^7^ Cancer Center Amsterdam, Department of Pulmonary Diseases, VU University Medical Center, Amsterdam, The Netherlands; ^8^ Cancer Center Amsterdam, Department of Pathology, VU University Medical Center, Amsterdam, The Netherlands; ^9^ Department of Pulmonary Diseases, Maastricht University Medical Center, Maastricht, The Netherlands; ^10^ Pivotal, Madrid, Spain; ^11^ Catalan Institute of Oncology, Hospital Germans Trias i Pujol, Barcelona, Spain; ^12^ Molecular Oncology Research (MORe) Foundation, Barcelona, Spain

**Keywords:** diagnostics, NSCLC, liquid biopsies, platelets, EML4-ALK

## Abstract

Purpose: Non-small-cell lung cancers harboring EML4-ALK rearrangements are sensitive to crizotinib. However, despite initial response, most patients will eventually relapse, and monitoring EML4-ALK rearrangements over the course of treatment may help identify these patients. However, challenges associated with serial tumor biopsies have highlighted the need for blood-based assays for the monitoring of biomarkers. Platelets can sequester RNA released by tumor cells and are thus an attractive source for the non-invasive assessment of biomarkers. Methods: EML4-ALK rearrangements were analyzed by RT-PCR in platelets and plasma isolated from blood obtained from 77 patients with non-small-cell lung cancer, 38 of whom had EML4-ALK-rearranged tumors. In a subset of 29 patients with EML4-ALK-rearranged tumors who were treated with crizotinib, EML4-ALK rearrangements in platelets were correlated with progression-free and overall survival. Results: RT-PCR demonstrated 65% sensitivity and 100% specificity for the detection of EML4-ALK rearrangements in platelets. In the subset of 29 patients treated with crizotinib, progression-free survival was 3.7 months for patients with EML4-ALK+ platelets and 16 months for those with EML4-ALK− platelets (hazard ratio, 3.5; *P* = 0.02). Monitoring of EML4-ALK rearrangements in the platelets of one patient over a period of 30 months revealed crizotinib resistance two months prior to radiographic disease progression. Conclusions: Platelets are a valuable source for the non-invasive detection of EML4-ALK rearrangements and may prove useful for predicting and monitoring outcome to crizotinib, thereby improving clinical decisions based on radiographic imaging alone.

## INTRODUCTION

Cancers driven by genetic alterations are likely to be sensitive to therapeutic inhibitors targeting the mutated pathway. For example, causative epidermal growth factor receptor (EGFR) mutations, present in approximately 16% of non-small-cell lung cancers (NSCLC), confer sensitivity to the EGFR tyrosine kinase inhibitors (TKIs) gefitinib, erlotinib and afatinib [1-3]. Rearrangements in the anaplastic lymphoma kinase (ALK) gene, occurring in 2-7% of NSCLC, also define a distinct molecular subtype of lung cancer [4]. The most common ALK rearrangement is an inversion that juxtaposes echinoderm microtubule-associated protein-like 4 (EML4) with ALK and produces the aberrant EML4-ALK fusion gene, with multiple chimeric variants, all of which encode the same portion of ALK but contain different truncations of EML4 [4]. ALK-rearranged tumors depend on ALK for growth and survival and show marked sensitivity to ALK inhibitors such as crizotinib, ceritinib, and alectinib [5-8]. However, despite initial responses, acquired resistance to these targeted therapies invariably leads to disease progression. Approximately one-third of patients with ALK-rearranged NSCLC relapse due to an acquired mutation within the ALK tyrosine kinase domain [9]. EML4-ALK amplification and bypass signaling, that re-establish activation of key downstream proliferation and survival signals despite inhibition of the original oncogene, are alternative mechanisms of resistance [10].

The assessment of biomarkers of resistance can facilitate the early identification of patients likely to relapse, ideally before radiographic imaging provides evidence of progression. However, while testing biopsied tumor tissue remains the current recommended method for mutation analysis, challenges associated with serial tumor biopsying, particularly in NSCLC, have spurred the search for non-invasive blood-based assays to allow frequent assessment of biomarkers as part of routine clinical care. Tumor cells can release RNA into the blood by a variety of microvesicle-dependent or -independent mechanisms, and several models have shown that platelets are crucial to tumor cell growth, spread, invasion, intravasation, migration, extravasation, and establishment of distant metastasis [11-13]. Using confocal microscopy and reverse transcription-polymerase chain reaction (RT-PCR), we have previously shown that platelets isolated from healthy individuals take up RNA-containing membrane vesicles from cancer cells [14]. Moreover, platelets isolated from patients with glioma and prostate cancer contained the cancer-associated RNA biomarkers EGFRvIII and prostate cancer antigen 3 (PCA3), respectively [14]. Since platelet RNA can be readily isolated and analyzed, platelets seem to be an attractive source for the non-invasive monitoring of biomarkers [15, 16]. However, to the best of our knowledge, the use of platelets to detect EML4-ALK rearrangements in patients with NSCLC has not yet been investigated.

We have assessed the feasibility of detecting EML4-ALK rearrangements in platelets and plasma from patients with NSCLC, with and without EML4-ALK-rearranged tumors, and from healthy controls. In addition, in a subset of patients treated with crizotinib we have examined the potential impact of EML4-ALK rearrangements in platelets on outcome. We have also monitored EML4-ALK rearrangements in platelets from one index patient throughout the course of treatment.

## RESULTS

### Detection of EML4-ALK rearrangements in platelets and plasma of NSCLC patients

This study was designed with three parallel objectives: firstly, to determine the sensitivity and specificity of detecting EML4-ALK rearrangements in platelets and plasma, correlated with matched tissue biopsies; secondly, to examine the potential impact of the EML4-ALK rearrangement in platelets on outcome to crizotinib; thirdly, to test the feasibility of monitoring a patient throughout treatment based on assessment of the EML4-ALK rearrangement in platelets.

**Figure 1:**
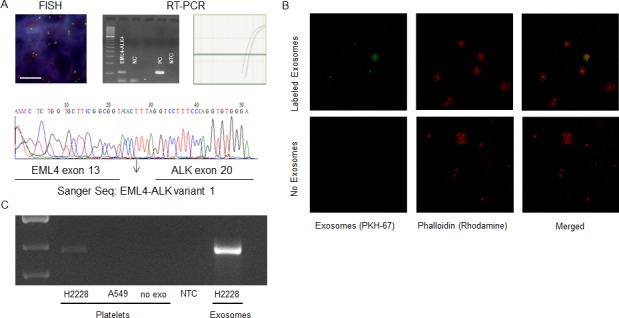
**A.** A representative EML4-ALK positive case by FISH in tissue and by RT-PCR in platelets (visual (left)- and quantitative (right)- analysis). Analyzed platelet samples were confirmed by Sanger sequencing of the RT-PCR products. The NSCLC human cell line H3122 served as positive control (PC). NC, negative control. **B.** Uptake of tumor-derived EML4-ALK RNA in platelets. Labeled exosome uptake in platelets, exosomes from the H2228 NSCLC cell line were labeled with PKH67. The exosome uptake into platelets was visualized as overlapping PKH67 labeled exosomes and labeled phalloidin (Rhodamine) in platelets. **C.** Positive transfer of EML4-ALK transcripts from H2228 exosomes into platelets. Exo, exosomes from positive control cell line; NTC, non-template control.

For the first objective, blood samples from 77 patients diagnosed with NSCLC between 2008 and 2014 were collected from public hospitals in The Netherlands (*n* = 49), Spain (*n* = 25), and the United States (*n* = 3). Thirty-eight patients had EML4-ALK-rearranged tumors, detected by fluorescence in situ hybridization (FISH), RT-PCR, or both (Figure 1A). Patient characteristics are shown in Supplementary Table 1. Thirty-five of these 38 patients received crizotinib - nine as first-line, 13 as second-line, and 13 as further lines of treatment - attaining an overall response rate (ORR) of 66% and median progression-free survival (PFS) of seven months (95% CI, 3.8 to 10.2).

Table 1 shows the detection rate of EML4-ALK rearrangements in paired tissue and blood samples. Plasma RNA was isolated from the blood samples of 32 patients, 14 of whom had EML4-ALK-rearranged tumors. EML4-ALK rearrangements were detected in plasma of three patients with EML4-ALK-rearranged tumors and in none of those without EML4-ALK rearrangements in their tumors, indicating 21% sensitivity and 100% specificity of the RT-PCR test in plasma RNA. Platelet RNA was isolated from the blood samples of 67 patients, 34 of whom had EML4-ALK-rearranged tumors. EML4-ALK rearrangements were detected in the platelets of 22 patients with EML4-ALK-rearranged tumors and in none of those without EML4-ALK rearrangements in tumor, indicating 65% sensitivity and 100% specificity of the RT-PCR test in platelet RNA. EML4-ALK rearrangements were not detected in platelets from any of the 21 healthy donors (Table 1).

**Table 1:** Detection of EML4-ALK rearrangements by RT-PCR in plasma and platelets from 77 NSCLC patients and 21 healthy controls

	EML4-ALK Rearrangement detected in Plasma	EML4-ALK Rearrangement detected in Platelets
NSCLC patients	*N* = 32*	*N* = 67*
EML4-ALK-rearranged tumor	3/14	22/34
EML4-ALK rearrangement not detected in tumor	0/18	0/33
Healthy controls	nd	0/21
Sensitivity	21%	65%
Specificity	100%	100%
Accuracy	66%	86%

nd, not determined

* Plasma and platelets were isolated in 32 and 67 samples respectively.

### Exosomes transfer RNA to platelets

Figure 1B and 1C demonstrate that exosomes released by cancer cells are vehicles capable of transferring tumor-derived EML4-ALK rearranged RNA into platelets, as shown by confocal microscopy and RT-PCR. Platelets were isolated from healthy donors and exosomes were isolated from H2228 EML4-ALK+ NSCLC cells. Isolated exosomes were labelled with PKH67 green fluorescent dye and subsequently incubated with the blood platelets. Confocal microscopy was used to confirm that the PKH67-labeled exosomes derived from H2228 NSCLC cells were internalized by the platelets (Figure 1B). RT-PCR was used to establish exosome-mediated transfer of tumor-derived EML4-ALK rearranged RNA from cancer cells to blood platelets. EML4-ALK RNA was detected by RT-PCR in platelets that were incubated with exosomes isolated from H2228 NSCLC cells and not in platelets incubated with exosomes from A549, an EML4-ALK − NSCLC cell line (Figure 1C).

### EML4-ALK rearrangements in platelets and outcome to crizotinib

Of the 35 crizotinib-treated patients, six had blood samples obtained at the time of progression only and were not included in the survival analyses, while 29 had blood samples obtained at baseline and/or during treatment before progression and were included in the analyses of PFS, overall survival and response. Median age of the 29 patients was 57 years (range, 37-81); 16 were never or former smokers; 24 had performance status (PS) 0-1; six had brain metastases (Supplementary Table 1).

**Figure 2:**
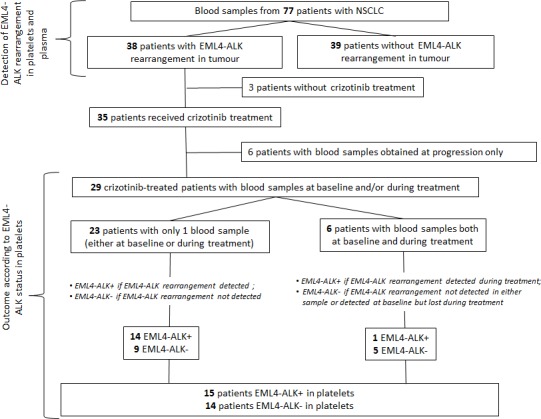
Flowchart showing the distribution of patients in the study Seventy-seven patients with NSCLC were included in the study testing the feasibility of detecting EML4-ALK rearrangements in platelets. A subset of 29 patients was included in the analysis of outcome according to EML4-ALK status in platelets.

Twenty-three of the 29 patients had only one blood sample available; six patients had two samples, obtained at baseline and during treatment. The EML4-ALK rearrangement was detected in platelets of 14 of the 23 patients with only one sample. These 14 patients were classified as EML4-ALK+, while the remaining nine were classified as EML4-ALK−. Of the six patients with two blood samples available, three had EML4-ALK rearrangement detected at baseline, but lost it during treatment and therefore classified as EML4-ALK−, for one case it was the reverse where EML4-ALK could be found during treatment, hence classified as EML4-ALK+. The remaining two samples were negative at both occasions. A total of 15 patients were classified as EML4-ALK+ and 14 as EML4-ALK− (Figure 2 and Supplementary Table S2).

**Figure 3:**
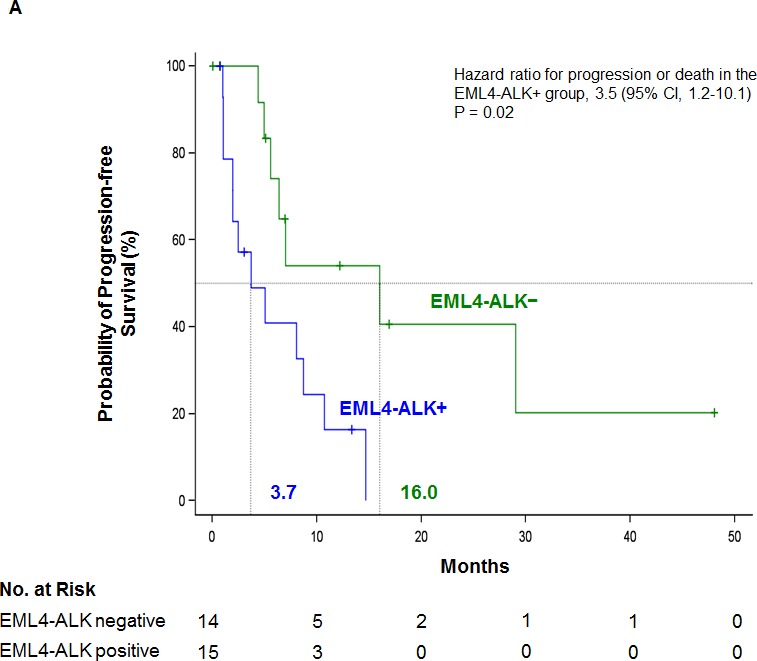

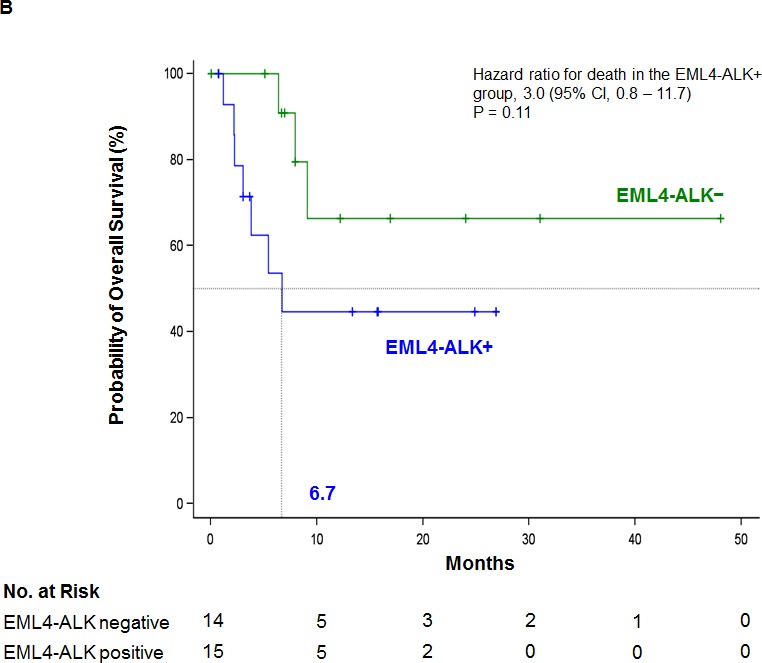

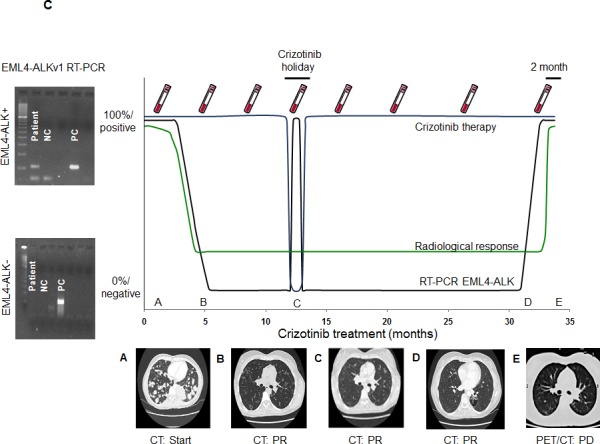
**Kaplan-Meier estimates of A.** PFS and **B.** overall survival according to status of the EML4-ALK rearrangement in platelets for 29 crizotinib-treated NSCLC patients. Tick marks on the survival curves indicate censored data. **C.** Longitudinal monitoring of crizotinib response in an index patient. Platelet EML4-ALK status was confirmed (shown to the left) at time points indicated by vials depicted above and radiological response depicted below corresponding to the time points represented by letter A-E. During one month rest from crizotinib due to appendicitis, EML4-ALK returned in platelets until the patient was back on crizotinib treatment. The EML4-ALK transcript was detected in platelets two months prior to radiographic disease progression. NC, negative control; PC, positive control; PR, Partial response; PD, Progressive disease; CT, computed tomography; PET, positron emission tomography

With a median follow-up of 13 months (range one to 48), PFS for all 29 crizotinib-treated patients was 7.0 months (95% confidence intervals [CI], 4.4 to 14.7), while it was 16 months (95% CI, 5.0 to not reached [NR]) for the 14 EML4-ALK− patients and 3.7 months (95% CI, 1.1 to 8.7) for the 15 EML4-ALK+ patients (*P* = 0.01) (Figure 3A). In the univariate analysis of PFS - including sex, age, PS, brain metastasis, number of previous lines of treatment, and EML4-ALK rearrangements in platelets - only EML4-ALK+ status was associated with shorter PFS (hazard ratio [HR], 3.5; 95% CI, 1.2 to 10.1; *P* = 0.02) (Figure 3A; Supplementary Table S3).

Median overall survival was NR (95% CI, 6.4 to NR) for all 29 crizotinib-treated patients, while it was NR (95% CI, 8.0 to NR) for the 14 EML4-ALK− patients and 6.7 months (95% CI, 2.3 to NR) for the 15 EML4-ALK+ patients (HR, 3.0; 95% CI, 0.8 to 11.7; *P* = 0.11) (Figure 3B). No variable was identified as a marker of overall survival in the univariate analysis (Supplementary Table S3). No significant differences in crizotinib response were observed between EML4-ALK+ and EML4-ALK− patients (Supplementary Table S4).

### Serial monitoring of EML4-ALK rearrangements in platelets of one representative patient

In order to test our hypothesis that the emergence of acquired resistance can be detected non-invasively in platelets before radiographic evidence of disease progression, we monitored a 37-year-old female non-smoker with NSCLC. EML4-ALK v1 was detected in her tumor and platelets at baseline in March 2012 (Figure 3C). She responded to crizotinib treatment and the EML4-ALK rearrangement was lost in her platelets. During a one-month interruption of crizotinib treatment due to appendicitis, the EML4-ALK rearrangement was detected in her platelets but was lost when she reinitiated crizotinib treatment. In June 2014, the EML4-ALK rearrangement was again detected in her platelets; however, positron emission tomography- computed tomography (PET-CT) did not show disease progression until two months later, in August 2014 (Figure 3C).

## DISCUSSION

In the present study, we have examined the use of platelets and plasma for the non-invasive assessment of EML4-ALK rearrangements and found that platelets are a promising biosource. Moreover, the detection and persistence of EML4-ALK rearrangement in platelets was significantly associated with shorter PFS to crizotinib. Interestingly, in the index patient, the re-appearance of the EML4-ALK rearrangement in platelets predicted resistance to crizotinib two months before radiographic progression. Platelets therefore can be considered alongside other promising biosources, like plasma, exosomes, circulating-free DNA and circulating tumor cells (CTCs), all of which have been proposed as alternative platforms for biomarker analysis (Supplementary Figure S1) [17-19]. In addition, Klement *et al*. have previously demonstrated selective uptake of tumor derived protein biomarkers in platelets [20, 21].

In the present study, plasma RNA had lower sensitivity than platelets (21% vs 65%) for the detection of EML4-ALK rearrangements by RT-PCR. The lower sensitivity in plasma may be attributed to a rapid degradation of free-circulating RNA molecules or low abundance of free-circulating exosomes containing rearranged EML4-ALK. Several groups have used CTCs for detection of biomarkers [22]. For example, Pailler *et al*., examined ALK rearrangements in CTCs of NSCLC patients and found four or more ALK-rearranged CTCs per 1 ml of blood, with 100% sensitivity and specificity [17]. Nevertheless, since CTCs typically constitute of a few cancer cell per tens of millions of normal nucleated cells in the blood, their use for the detection of EML4-ALK rearrangements must rely on novel CTC detection strategies. Circulating-free DNA has limitations when it comes to large rearrangements, since finding the chromosomal brake-point needs extensive deep sequencing of the genomic DNA. Unfortunately, platelets are not yet stored routinely and prospective collection is often required to analyze platelet RNA. Another disadvantage of blood platelets could be their potential reaction towards certain therapies, resulting in reduced platelet counts in the blood and potential platelet activation. However, in direct comparison with the other liquid biosources for the detection and monitoring of EML4-ALK rearrangements, platelets are a valid and competitive biosource when it comes both to detection and response to therapy, as well as ease of extraction and rapid readout of the assays. The utility of our EML4-ALK test in therapy monitoring was illustrated in our index patient from whom we monitored EML4-ALK rearrangements in platelets throughout the course of her treatment. This index case exemplifies the use of serial monitoring of EML4-ALK rearrangements in platelets to predict progression, thereby providing physicians with an early window of opportunity to modify treatment and switch to a second-generation ALK inhibitor, such as ceritinib or alectinib.

EML4-ALK rearrangements in platelets are a direct readout of the effect of crizotinib on EML4-ALK-rearranged NSCLC cells. Adequate crizotinib-mediated cell eradication results in reduced release of EML4-ALK transcripts into circulation, and hence the loss of platelet EML4-ALK transcripts. By contrast, crizotinib resistance causes continuous production and release of EML4-ALK transcripts. The source of DNA in the blood of cancer patients are cells that disintegrate by apoptosis and/or necrosis in expanding tumor tissue [23]. In contrast RNA released into the blood by microvesicle-dependent or -independent mechanisms is functionally active in recipient cells, suggesting that tumor cells can exploit circulating RNA as a means to ‘communicate’ with their regional or distal environment [24]. Therefore, an expressed biomarker (e.g. EML4-ALK RNA molecules) will tell us more about cellular dysfunctions than biomarkers derived from apoptotic or necrotic cells (e.g. fragmented DNA). This further supports non-invasive RNA based blood tests for the monitoring of ongoing alterations within the tumor while responding to therapy.

Although patients in our study with EML4-ALK+ platelets had dismal prognosis, our finding can also lead to potentially beneficial opportunities for treatment in this subgroup of patients. Recruitment of platelets by tumor cells is regulated by signaling molecules released by the tumor or its metastases. One of these molecules is Aggrus/podoplanin, a protein that attracts platelets expressing the CLEC-2 receptor [13]. Blocking the binding of Aggrus/podoplanin to CLEC-2-expressing platelets could be a novel therapeutic approach with benefit for EML4-ALK+ patients.[25] ALK has recently been shown to regulate the production of vascular endothelial growth factor A (VEGFA) in anaplastic large-cell lymphoma and EML4-ALK-rearranged NSCLC, and the anti-VEGFA antibody bevacizumab strongly impaired the growth of anaplastic large-cell lymphoma in mouse xenografts [26]. Activated platelets release several growth factors, including VEGF and, therefore, the subset of EML4-ALK+ patients could likely benefit from anti-angiogenic therapy [13, 26].

## MATERIALS AND METHODS

### Cell lines

The NSCLC human cell lines H3122 and H2228 were kindly provided by Dr. Daniel Costa (Department of Medicine, Harvard Medical School, Boston, MA) and served as positive controls for the variant 1 and the shorter variant 3 of EML4-ALK transcript, respectively. The NSCLC human A549 cell line was obtained from the American Type Culture Collection (ATCC) collection.

### Isolation of platelet and plasma RNA

Platelets and plasma were isolated from the same sample of whole blood in 6ml purple-cap BD Vacutainers containing EDTA anti-coagulant by standard centrifugation. The cells and aggregates were removed by centrifugation at room temperature for 20 min at 120g, resulting in platelet-rich plasma. The platelets were isolated from the platelet-rich plasma by centrifugation at room temperature for 20 min at 360g, after which the plasma was centrifuged at 360g for 10 min and frozen. The platelet pellet was collected in 30 μl RNAlater (Life Technologies, Carlsbad, CA) and frozen at −80°C for further use. Platelets and plasma were frozen in parallel. Platelet RNA and plasma RNA were isolated using either an Agencourt Formapure (Agencourt Biosciences, Beverly, MA), miRvana RNA isolation kit (Life Technologies), or Trizol (Life Technologies), according to the manufacturers' instructions. The concentration and quality of the platelet RNA was determined with an Agilent 2100 Bioanalyzer with total RNA Pico chip (Agilent, Santa Clara, CA). Platelet RNA of sufficient quality (Bioanalyzer RIN values >7 and/or distinctive rRNA curves) was subjected to biomarker analysis. The quality of plasma RNA could not be measured and only quantitation of RNA input was used.

### Isolation and exosome uptake

Exosomes were isolated by ultracentrifugation using cell culturing medium from EML4-ALK positive (H2228) and wild type control (A549) NSCLC cell lines. The medium and plasma was first cleared of debris by centrifugation; twice at 500xG for 10 minutes, followed by twice at 2,000xG for 15 minutes, and lastly twice at 10,000xG for 30 minutes. The exosomes in the cleared sample were pelleted at 100,000xG for 60 minutes. The exosomes were washed once in PBS before either being labeling for uptake experiments or DNAse-I treated (4 units; Promega) for 15min before lysis in miRVANA lysis buffer (Ambion) for RNA extraction (according to manufacturer's recommendations) for mutation detection experiments. Exosome uptake was analyzed using PKH67-labeled (Sigma-Aldrich) H2228 microvesicles and the LSM-710 confocal microscope system with the ZEN 2010 software (Carl Zeiss) and a 63x oil immersion objective (Carl Zeiss) as previously described [14]. Platelets were stained with rhodamine phalloidin (Invitrogen) to indicate platelet structure and analyzed for exosomal uptake by the presence of green fluorescent dye PKH67 (Sigma-Aldrich). EML4-ALK rearranged RNA within platelets was shown by PCR on reverse transcribed RNA with the same setup as previously described.

### RT-PCR analysis of EML4-ALK rearrangements

At thromboDx, quantitative PCR Taqman assays from Life Technologies (Hs03654556, Hs03654557, and Hs03654558) were used for detection of the three most common EML4-ALK variants (v1 [e13:a20], v2 [e20:a20], and v3 [e6:a20], with breakpoints at EML4 exons 13, 20, and 6 and at ALK exon 20, respectively), according to the manufacturer's instructions. Quantitative PCR was run on the ABI7500 (Applied Biosystems, Foster City, CA) for 60 cycles in a standard TaqMan program with TaqMan human GAPDH control reagents (Life Technologies, 402869) as input control. EML4-ALK breakpoints were confirmed by Sanger sequencing of RT-PCR products, using the Big-Dye 1.1 sequencing kit (Applied Biosystems). At Pangaea Biotech, EML4-ALK was amplified by PCR and visualized in agarose gels. RNA was converted to cDNA by superscript III (Life Technologies) and amplified using 40 ng of cDNA and 1U Platinum Taq polymerase (Invitrogen) in a 20 μl reaction. Specific forward and reverse primers were used for the three most common EML4-ALK variants (Supplementary Table S5). EML4-ALK breakpoints were confirmed by Sanger sequencing of RT-PCR products, using the Big-Dye 1.1 sequencing kit (Applied Biosystems).

### Statistical analyses

All participating centers sent samples in a non-consecutive manner, and there was no stratification according to sex, Eastern Cooperative Oncology Group (ECOG) PS, smoking history, or prior treatment. PFS was calculated from the start of crizotinib treatment until first observation of disease progression by serial CT and/or PET-CT. Overall survival was calculated from the start of crizotinib treatment until death from any cause. Median PFS and overall survival, with their 95% CIs, were estimated with the Kaplan-Meier method and compared using the non-parametric log-rank test. The association between each potential prognostic factor and PFS or overall survival was assessed with a univariate Cox proportional-hazard regression model. All statistical analyses were performed with the use of SAS V9.3. Significance was set at P < 0.05.

### Study oversight

This study was conducted in accordance with the principles of the Declaration of Helsinki. Written and/or oral informed consent was obtained from all patients and documented. Approval was obtained from the institutional review board and the ethics committee at each hospital. The drug used in the study was purchased from the manufacturer, who had no role in the study. The data were collected and analyzed by the senior authors in conjunction with all authors. The corresponding author wrote the first draft of the manuscript. All the authors approved the final version of the manuscript, agreed with the decision to submit the manuscript for publication and vouched for the accuracy of the data and analyses reported.

## SUPPLEMENTARY MATERIAL FIGURE AND TABLES


