# Supplementary material for: Rearranged EML4-ALK fusion transcripts sequester in circulating blood platelets and enable blood-based crizotinib response monitoring in non-small-cell lung cancer
Source: Oncotarget. 2015 Nov 2;7(1):1066–75. doi: 10.18632/oncotarget.6279 (PMC4808052; doi:10.18632/oncotarget.6279)
Supplement: Supplementary file 1 [file oncotarget-07-1066-s001.pdf]

Rearranged EML4-ALK fusion transcripts sequester in circulating blood platelets and enable blood-based crizotinib response monitoring in non-small-cell lung cancer

Supplementary Material

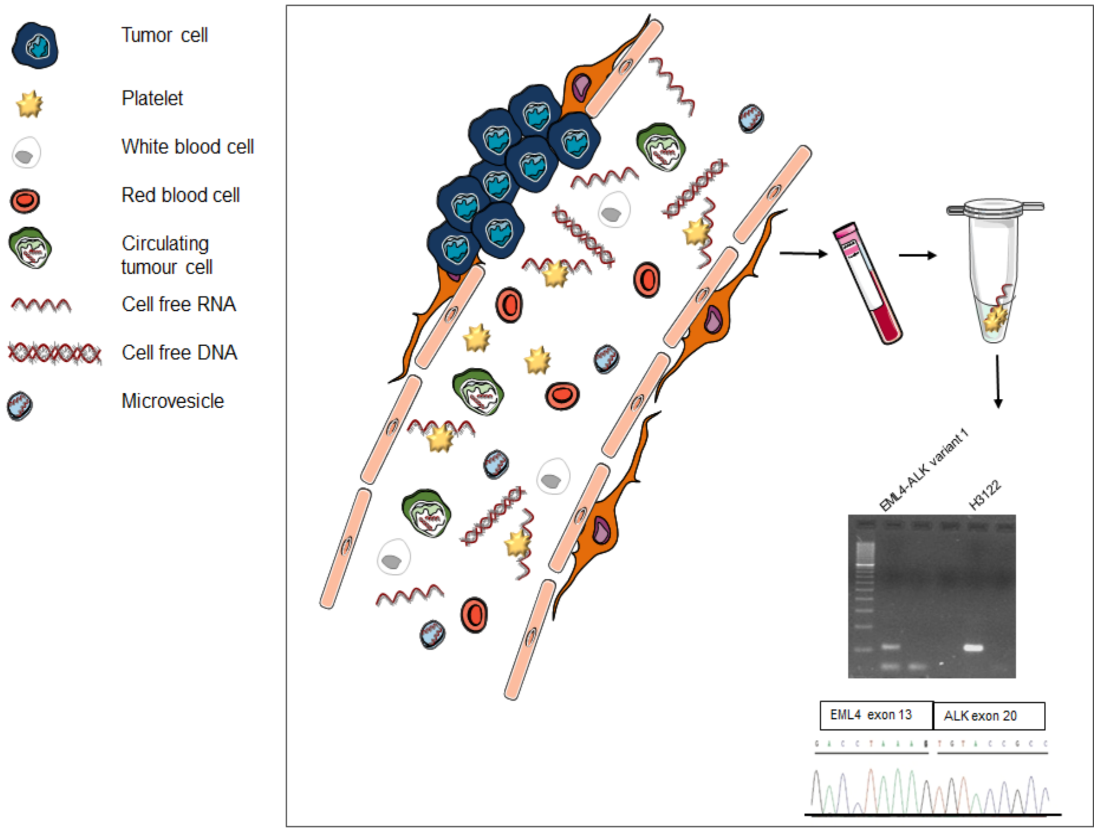

Figure S1: Platelets contain tumor-derived RNA biomarkers. Tumour-related circulating-free DNA, RNA or proteins are released by tumour cells through various events such as apoptosis or necrosis and circulate in the blood of cancer patients. Platelets are capable of taking up circulating-free RNA. Indeed tumour cells have the ability to 'educate' platelets, loading them with tumour-derived RNA. With a half-life of approximately ten days, platelets are a promising source for the non-invasive detection of EML4-ALK rearrangements. The agarose gel shows the EML4-ALK rearrangement in platelets isolated from an EML4-ALK-rearranged NSCLC patient.

Table S1: Clinicopathological characteristics of 38 patients with EML4-ALK-rearranged NSCLC tumours and of the subset of 29 patients treated with crizotinib included in the analyses of PFS and overall survival. There were no significant differences between the characteristics of the two groups.

Characteristic	All Patients N=38 N (%)	Crizotinib-Treated Patients N=29 N (%)
Age-years		
Median (range)	60 (37-81)	57 (37-81)
Sex		
Female	13 (34)	10 (34)
Male	25 (66)	19 (66)
Smoking history		
Never smoked	18 (47)	11 (38)
Former smoker	6 (16)	5 (17)
Current smoker	8 (21)	8 (28)
Not reported	6 (16)	5 (17)
ECOG PS		
0	6 (16)	4 (14)
1	22 (58)	20 (69)
2-3	4 (10)	3 (10)
Not reported	6 (16)	2 (7)
Histology		
Adenocarcinoma	37 (97)	29 (100)
Large-cell carcinoma	1 (3)	0
Brain metastasis		
No	23 (61)	23 (79)
Yes	13 (34)	6 (21)
Not reported	2 (5)	0
Previous lines of treatment		
0	9 (24)	9 (31)
1	13 (34)	10 (35)

2	5 (13)	5 (17)
>2	8 (21)	5 (17)
Not reported	3 (8)	0 (0)
Detection of EML4-ALK rearrangement in tumour		
FISH	24 (63)	18 (62)
RT-PCR	4 (11)	2 (7)
FISH & RT-PCR	10 (26)	9 (31)
Treatment		
Crizotinib	35 (92)	29 (100)
Other	3 (8)	0

ECOG, Eastern Cooperative Oncology Group; PS, performance status; FISH, fluorescence in situ hybridization; RT-PCR, reverse transcription polymerase chain reaction

Table S2: Detection of EML4-ALK rearrangements in platelets of 29 crizotinib-treated patients

Patient ID		EML4-ALK in platelets		Patient status*
		At baseline	During treatment	
Patients with blood samples obtained at baseline				
1	V019	Detected		EML4-ALK+
2	B665	Detected		EML4-ALK+
3	B849	Detected		EML4-ALK+
4	V0132	Detected		EML4-ALK+
5	V068	Detected		EML4-ALK+
6	V045	Detected		EML4-ALK+
7	VU125	Not detected		EML4-ALK−
8	V060	Detected		EML4-ALK+
9	B1022	Detected		EML4-ALK+
10	V063	Not detected		EML4-ALK−
11	M003	Not detected		EML4-ALK−
12	V077	Detected		EML4-ALK+
13	V065	Not detected		EML4-ALK−
14	V0103	Not detected		EML4-ALK−
15	V053	Not detected		EML4-ALK−
16	V038	Detected		EML4-ALK+
17	V078	Detected		EML4-ALK+
18	V086	Not detected		EML4-ALK−
19	V072	Detected		EML4-ALK+
Patients with blood samples obtained during treatment				
20	M001		Detected	EML4-ALK+
21	V0101		Not detected	EML4-ALK−
22	M002		Detected	EML4-ALK+
23	B295		Not detected	EML4-ALK−
Patients with blood samples obtained at baseline and during treatment				
24	V0106	Not detected	Not detected	EML4-ALK−
25	B1008	Detected	Not detected	EML4-ALK−

26	V083	Not detected	Detected	EML4-ALK+
27	B739	Not detected	Not detected	EML4-ALK−
28	V064	Detected	Not detected	EML4-ALK−
29	B676	Detected	Not detected	EML4-ALK−

*The 23 patients with blood samples obtained only at baseline or only during treatment were classified as EML4-ALK+ if the EML4-ALK rearrangement was detected in their blood samples. The remaining six patients, who had samples available both from baseline and during treatment, were classified as EML4-ALK+ if the EML4-ALK rearrangement was detected either in both samples or only in the one obtained during treatment. A negative EML4-ALK blood sample status obtained during treatment classified the patient as EML4-ALK-.

Table S3: Univariate analyses of PFS and overall survival in 29 crizotinib-treated patients

	PFS		Overall Survival	
	Hazard Ratio (95% CI)	P	Hazard Ratio (95% CI)	P
Sex				
Female	1.0 (ref)		1.0 (ref)	
Male	0.9 (0.4-2.4)	0.88	0.7 (0.2-2.6)	0.61
Age				
<57 years	1.0 (ref)		1.0 (ref)	
≥57 years	0.5 (0.2-1.4)	0.19	0.4 (0.1-1.5)	0.19
ECOG PS				
0-1	1.0 (ref)		1.0 (ref)	
≥2	1.0 (0.2-4.5)	0.95	1.4 (0.2-11.3)	0.75
Not reported	1.5 (0.3-6.5)	0.62	1.0 (0.1-7.3)	0.93
Brain metastasis				
No	1.0 (ref)		1.0 (ref)	
Yes	1.9 (0.7-5.5)	0.24	1.6 (0.4-6.1)	0.51
No. of previous lines of treatment				
0	1.0 (ref)		1.0 (ref)	
1	1.3 (0.4-4.1)	0.64	3.7 (0.4-31.9)	0.23
≥2	0.7 (0.2-2.5)	0.64	2.5 (0.3-22.1)	0.42
EML4-ALK patient status*				
EML4-ALK−	1.0 (ref)		1.0 (ref)	
EML4-ALK+	3.5 (1.2-10.1)	0.02	3.0 (0.8-11.7)	0.11

PFS, Progression free survival; ECOG, Eastern Cooperative Oncology Group; PS, performance status. *The 23 patients with blood samples obtained only at baseline or only during treatment were classified as EML4-ALK+ if the EML4-ALK rearrangement was detected in their blood samples. The remaining six patients, who had samples available

both from baseline and during treatment, were classified as EML4-ALK+ if the EML4-ALK rearrangement was detected either in both samples or only in the one obtained during treatment. A negative EML4-ALK blood sample status obtained during treatment classified the patient as EML4-ALK-.

Table S4: Response to crizotinib treatment in 29 patients according to the EML4-ALK rearrangement in platelets. There was no significant difference between EML4-ALK+ and EML4-ALK− patients (P=0.35).

	EML4-ALK− Patients<br>N=14<br>N (%; 95% CI)	EML4-ALK+ Patients<br>N=15<br>N (%; 95% CI)	All Patients<br>N=29<br>N (%; 95% CI)
Partial Response	11 (79%; 49 to 95)	9 (60%; 32 to 84)	20 (69%; 49 to 85)
Stable Disease	3 (21%; 5 to 51)	3 (20%; 4 to 48)	6 (21%; 8 to 40)
Progressive Disease	0	3 (20%; 4 to 48)	3 (10%; 2 to 27)

Table S5: Primers used for the detection of EML4-ALK rearrangements at Pangaea Biotech, SA, Barcelona

EML4-ALK rearrangements	Primers	
	Forward	Reverse
Variant 1 (e13:a20)	5’ TCC TTA CTG GAG ACT CAG GTG GAG T 3’	5’ TTG CAG CTC CTG GTG CTT C3’
Variant 2 (e20:a20)	5’ ACA TCA CAC ACC TTG ACT GGT CC 3’	5’ AGC TTG CTC AGC TTG TAC TCA GGG 3’
Variant 3 (e6:a20)	5’ ACCTTCAAC ACCCAAATTAATACCA 3’	5’ TGTACTCAGGGCTCTGCAGCT 3’
